# Single Locus Maintains Large Variation of Sex Reversal in Half-Smooth Tongue Sole (*Cynoglossus semilaevis*)

**DOI:** 10.1534/g3.116.036822

**Published:** 2016-12-21

**Authors:** Li Jiang, Hengde Li

**Affiliations:** Centre for Applied Aquatic Genomics, Chinese Academy of Fishery Sciences, Beijing 100141, China

**Keywords:** sex reversal, genome-wide association study, half-smooth tongue sole, *FBXL17*

## Abstract

Sex determination is a fundamental biological process for individual sex development and population sex ratios. However, for some species, the primary sex might be altered during development, and individuals can develop into the opposite sex. Sex reversal may happen in insects, reptiles, amphibians, and fishes. In half-smooth tongue sole (*Cynoglossus semilaevis*), some genetically female fish irreversibly reverse to pseudomales, resulting in higher costs in aquaculture owing to a lower growth rate of male fish during a 2-yr growth period. Here, we identified a locus with large controlling effect on sex reversal in the half-smooth tongue sole through genome-wide association study with high-density single nucleotide polymorphisms (SNPs). This SNP is located at the third intron of the F-box and leucine rich repeat protein 17 (*FBXL17*) gene on the Z chromosome, and it has two alleles, A and T. Genetic females with Z^A^W genotypes will never reverse into phenotypic males, but those with Z^T^W genotypes can sometimes undergo sex reversal. This SNP explains 82.7% of the genetic variation, or 58.4% of the phenotypic variation. Based on our results, a reproductive management program could be developed to improve the phenotypic female ratio in aquaculture, and elucidate the mechanism of sex reversal in half-smooth tongue sole. We expect that these findings will have a substantial impact on the population management in many harvested species where sex reversal occurs.

Sex determination is a fundamental biological process for individual sex development and population sex ratios. Usually, mammals and birds show a genetic chromosomal form of sex determination, referred to as genetic sex determination (GSD). Mammals have an XY male/XX female system, and birds have a ZZ male/ZW female system. The GSD system is very stable for mammals and birds, and, therefore, it is thought sex is determined once the egg has been fertilized. However, for some species, the primary sex may be altered during development, and individuals can develop into the opposite sex, leading to sex ratios in populations departing from 1:1. This phenomenon is known as sex reversal, and may happen in insects (Narita *et al.* 2007), reptiles (Quinn *et al.* 2007), amphibians (Wallace *et al.* 1999), and fishes (Nagahama 2005).

Ecologists considered sex reversal a successful, adaptive, and reproductive strategy for animals in evolution (Shapiro 1987; Ghiselin 1974). Many studies reported environmental factors, such as temperature, hormones, and pH values, critically affect sex reversal, of which temperature has been the most studied. Sex differentiation usually occurs during the early stages of development; cold treatment on uncleaved eggs to those undergoing early metamorphosis did not affect the sex ratio, whereas high temperatures tended to induce sex reversal from female to male, *e.g.*, *Triturus cristatus* (Wallace *et al.* 1999) and half-smooth tongue sole (*Cynoglossus semilaevis*) (S. Chen *et al.* 2014), or from males to females, *e.g.*, *Pogona vitticeps* (Holleley *et al.* 2015).

Temperature-dependent sex determination (TSD) and GSD are not mutually exclusive dichotomies (Sarre *et al.* 2004); many species have stable differentiated sex chromosomes, but also show temperature interference, indicating sex reversal is determined by the interaction of genes and the environment. Evidence of sex reversal in *P. vitticeps* indicated a transition from GSD to TSD (Holleley *et al.* 2015), which showed the genetic background as critical factors. In other examples, a group of genes have been found to be involved in TSD in turtles (Chojnowski and Braun 2012); and a study in medaka showed that an autosomal locus and sex-determining region on the Y chromosome contributed simultaneously to sex reversal (Shinomiya *et al.* 2005; Kato *et al.* 2010, 2011).

*C. semilaevis* is a valuable commercial fish, which distributed along the coast of the Yellow and Bohai Seas of China. The primary sex of *C. semilaevis* is determined by the female heterogametic sex determination system (ZW♀/ZZ♂). The females and males have considerably different growth rates, where adult females are 2–4 times larger than adult males in size (Chen *et al.* 2009). Therefore, female fish are preferred in *C. semilaevis* aquaculture; however, some genetic females can reverse into phenotypic males in the early stages from around d 50 to 90. In *C. semilaevis*, sex reversal is also sensitive to high temperatures, and the number of sex reversed or pseudomale fish increases with increasing temperature (S. Chen *et al.* 2014), and, even in normal conditions (22°), it is still easy to find a large portion of pseudomale fish.

Sex reversed or pseudomale fish have a similar growth rate to normal males, resulting in increased costs for farmers than those for females with higher growth rates. To date, the female ratios in most farms are around 20%; hence, controlling the occurrence of sex reversal, or improving the female ratio, is the most important problem for sustainable aquaculture. Some management techniques have been developed to utilize pseudomales to produce all-female offspring or improve female ratios for ZW sex-determination fish (Piferrer *et al.* 2012), but it is not feasible for half-smooth tongue sole, because WW fish cannot survive in early development, and genetic offspring of pseudomales tends to reverse more than those of normal males (S. Chen *et al.* 2014).

In tongue sole aquaculture, most farms can control the temperature relatively well to 22°, but not all genetic female offspring of the same family, and under the same environment, reversed to pseudomales; therefore, this dichotomy implies the genetic difference between reversed and nonreversed fish, and it was speculated that genes on the Z chromosome may contribute to sex reversal (S. Chen *et al.* 2014). Therefore, it is necessary to investigate if there are variants controlling sex reversal, and if we can genetically control sex reversal in tongue sole aquaculture. In the present study, we obtained the sex reversal phenotype using tissue sections combined with molecular markers, and performed a genome-wide association study (GWAS) to detect the genetic locus associated with sex reversal using an efficient generalized linear mixed model (Kang *et al.* 2010).

## Materials and Methods

### Materials

In March 2013, six adult females and 11 phenotypic males of half-smooth tongue sole were randomly selected and tagged as the parents, which were cultivated in Dongying, Shandong Province, China. These parental fish were raised in the same pond for reproduction. The larvae were transferred to another pond 36 hr after hatching. Since sex reversal of half-smooth tongue sole usually happens in the first 90 d in early stage, so this experiment was designed to last for 90 d at a constant temperature of 22° to avoid additional environmental effects. Thereafter, 268 fry were randomly selected for DNA extraction, parentage analysis, and genetic and phenotypic sex detection. Finally, 115 genetic females were used for GWAS. In April 2014, 399 fish raised under the same conditions as those in 2013, with no parental information, were randomly harvested from the same farm for confirmation of our detected polymorphism associated with sex reversal.

### Phenotypes

Fins and gonads were sampled, and fins were used for DNA extraction. The genetic sex was determined using the method of Chen *et al.* (2009), and all the samples were assigned parents with 21 pairs of SSR primers (Song *et al.* 2012) (Table 1) using Cervus 3.0 software (Slate *et al.* 2000) with a Maximum Likelihood approach; only individuals with 99.99% liability were reported. Thereafter, gonads of genetic females were used for phenotypic sex determination by tissue section (Supplemental Material, File S1).

**Table 1:** Primers for parentage analysis

Primer Name*^a^*	Primer Sequence (5′–3′)	Code
1-F	CCCCAGAGCAGGTTCAATC	JN902297
1-R	GCGCTAACAGGTGTTCAAACA	
2-F	CGTCAGTGGTTACAGGCAAC	JN902320
2-R	CAATGACACCCTTGTCGTTCG	
3-F	ACACCGTCATAGTGTTTGGCA	JN902351
3-R	ACAGCTTGGCAGTGTTCTCT	
4-F	GACTCTTCATCGACTGGGAGAC	JN902892
4-R	GCTGGGCGATAGAGAGACAT	
5-F	TCAAGGACTGTGTTACGAAGGAG	JN902384
5-R	GAACCCAGATGAGCTGGATGA	
8-F	AGTCTGGCCTGGAGTTTGT	JN902469
8-R	AGCTACCAGGTGAGGAGC	
10-F	TGTTGTTTCCTGTCTCCGTCTG	JN902541
10-R	GTCACAGCTGCAGTCACAC	
11-F	CTGTGGCGCTATGCTCAATTAC	JN902870
11-R	CGGTGTCTGTGGATCTGTTCT	
12-F	AGGAGAACAGGTCAGTCATACGA	JN902640
12-R	CTCGTTCCAAACTCTCCTCCA	
15-F	TTTATCTCAGCCAGCAGCAA	HM060584
15-R	CCACGGACAACGCACTTTA	
16-F	GGACCTGCTGCTGTTATGTC	EU907031
16-R	CTGCACAGGAGTGAACTGTG	
17-F	AGCTGGAGCTACCACTACCT	JN902723
17-R	CGGACCATGCACGTATTGAAC	
18-F	GGCTGTTAATCTGGTCACA	JN902750
18-R	CAGATAATTGGTCCCCTGAA	
20-F	TGCTGCAGTGAGGGTTTCAC	JN902776
20-R	CCAGAAAACACTGGCAGCTCT	
21-F	AGCCAGAGTCTCACACATCG	JN902795
21-R	GCGTGCTACAAAGAACAGACG	

a The *T_m_* for all pair of primers is 56°.

Gonad tissues samples were immediately placed into Bouin’s fixation solution overnight. The fixed tissues were dehydrated in steps from 70, 80, and 90, to 95% ethyl alcohol for 1 hr each, respectively, and finally in 100% ethyl alcohol for 40 min. Gonad tissues were cleared in solution (xylene:ethyl alcohol, 1:1) for 40 min, then in xylene for 30 min. The tissues were treated with a paraffin solution (xylene:paraffin, 1:1) for 1 hr, then pure paraffin for 1 hr twice. The tissues were embedded with paraffin for sectioning. Splices of 6 µm were produced using a Leica 2200 tissue splicer. Dewaxing was carried out in pure xylene 15 min twice, and then samples were placed in 100% benzene alcohol for 5 min. Samples were rehydrated in 100, 90, 80, 70, 50, and 30% ethyl alcohol for 2 min each, respectively, then in deionized water for 3 min twice. The samples were stained using HE solution. The HE solution included four types: (A) 5 g hematoxylin dissolved in 50 ml ethyl alcohol; (B) 100 g alum dissolved in 1000 ml water; (C) solution A and B were mixed together and heated for 2 min, and 2.5 g mercuric chloride was added, then the solution was left to stand overnight, then the solution was filtered; and (D) 10 g eosin was dissolved in 1000 ml ethyl alcohol.

Samples were stained in A solution for 5–10 min, then in water for 10 min until the water became blue, then for another 3 min in fresh water; next they were washed in hydrochloric acid solution (1 ml concentrated hydrochloric acid dissolved in 99 ml 70% ethyl alcohol) for several seconds until the samples became light red. Samples were washed in water for 3 min until the water became blue. The samples were then placed in gradient ethyl alcohol for 2 min respectively for each step, from 30, 50, and 70, and finally 80%. The samples were then stained in eosin solution for ∼20 sec, and placed in the gradient ethyl alcohol for 2 min per step, from 90, 95, 100, and 100% ethyl alcohol, then benzene alcohol for 2 min, and xylene for 2 min twice.

The samples were observed and analyzed using an Olympus microscope.

### Genotypes

One hundred and fifteen genetic females were genotyped using the 2b-RAD method from the Oebiotech Co. Ltd. (Shanghai, China). The 2b-RAD libraries were prepared with *Bsa*XI following the reported protocol (Wang *et al.* 2012), and were subjected to single-end sequencing using an Illumina HiSeq2500 platform. Using sequencing, a total of 612,818,007 reads was produced, averaging 5,328,852 reads per sample. The 2b-RAD genotyping were performed with the RADtyping program v1.5 (Fu *et al.* 2013). Reads with no restriction sites, or containing ambiguous base calls (*N*), long homopolymer regions (>10 bp), excessive numbers of low quality positions (>10 positions with quality of <20) were removed. Finally, 138,666 unique tags were obtained, an average level of coverage was 16× per sample. Totally 66,563 SNP genotypes were obtained and mapped to *C. semilaevis* genome (S. Chen *et al.* 2014) with SOAP2 (Li *et al.* 2009). The SNPs with minor allele frequencies of <5% or a call rate of <0.90 were discarded, and the remaining missing data were imputed through linkage disequilibrium with 10 closest neighboring markers (see File S2 for the source code). After the quality control, the final genotypic data (File S3 and File S4) in the analysis consisted of 17,618 SNPs without missing genotypes (Table 2).

**Table 2:** The SNP information

Chromosome	Number of SNPs	Span of SNPs (Mb)
1	1573	34.47
2	846	20.05
3	747	16.24
4	876	19.90
5	977	19.17
6	882	18.82
7	734	13.76
8	1276	30.11
9	829	19.60
10	905	20.92
11	841	20.43
12	748	18.30
13	806	21.79
14	1081	28.83
15	734	19.88
16	792	18.65
17	686	16.46
18	532	14.94
19	677	17.72
20	705	15.15
W	110	16.17
Z	261	21.40

### Statistical analysis

Sex reversal was considered a binomial trait, and the incidence of sex reversal was recorded as one, and the others as zero. We used a logistic animal model to perform an association analysis:

logit(y)=μ+bm+g+e (1)

where logit(y)=p/(1−p); *p* is the frequency of sex reversal; *m* is SNP genotype, and was taken as a fixed effect; *b* is the allele substitution effect; *g* is the additive effect and follows the distribution $N\left(0,K\sigma_{g}^{2}\right);$ **K** is the realized genetic relationship matrix; and *e* is the random error and follows the distribution of $N\left(0,\sigma_{e}^{2}\right).$ Construction **K** follows these procedures, (1) code the genotype matrix **M** for each marker, 0 for homozygotes, 1 for heterozygote, and 2 for the other homozygote; (2) standardize **M** for each SNP as **Z**, (3) calculate **K** as $ZZ^{\prime }/n_{m},$ where *n_m_* is the number of markers. An association analysis of each SNP was conducted by comparing the full to the null model:

logit(y)=μ+g+e (2)

and the −2*log-likelihood test statistics approximately followed the Chi-square distribution with a freedom of degree of 1. To improve the computation speed, a method similar to that of the efficient multi-locus mixed model (Segura *et al.* 2012) was used. First, DMU (Madsen and Jensen 2002) was employed to estimate the variance components, $\sigma_{g}^{2}$ and $\sigma_{e}^{2},$ then the linear mixed model was transformed to a simple linear regression through the Cholesky decomposition of the phenotypic (co)variance structure **V** as the following equation:

y*=bm*+e* (3)

where $y*=V^{-\left(1/2\right)}*\mathrm{logit}\left(y\right),$ $M*=V^{-\left(1/2\right)}*M,$ and $V=K\text{*}\sigma_{g}^{2}+I*\sigma_{e}^{2},$ m* is the corresponding SNP column of the M* matrix. After a genome scan, the threshold value was determined (Piepho 2001), and, once a genetic locus was detected, the corresponding confidence interval was calculated with Li’s method (Li 2011).

### Data availability

File S1 is phenotypic file containing id, sire, dam, genetic sex, phenotypic sex, and incidence of sex reversal. File S2 contains the source code for miss genotype imputation. File S3 contains genotypes (coded as 0, 1 and 2) of 115 samples, and File S4 is the SNP information.

## Results

### Sex determination and parentage analysis

**Figure 1:**
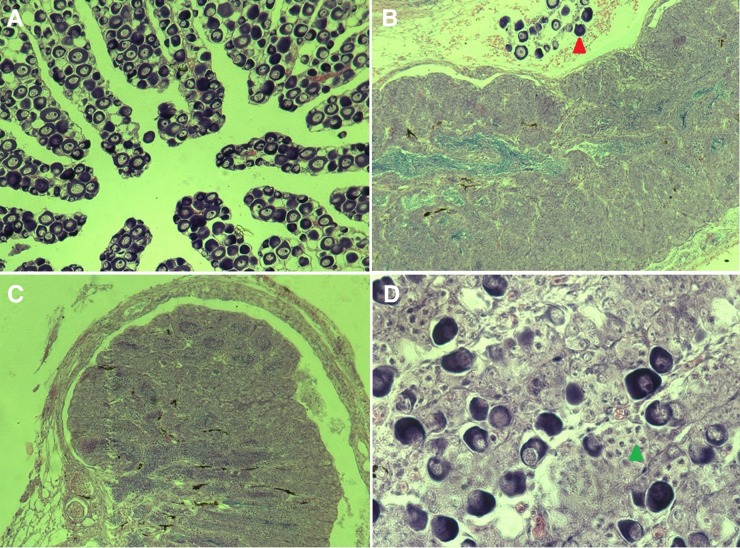
Sex differentiation of the half-smooth tongue sole for pseudomales. (A) Ovary and oocytes (×100). (B) Chimeric gonad during the late process of sex reversal from ZW female to pseudomale, mainly composed of spermatangia; the red triangle indicates the oocytes (×100). (C) Testis and sperm cells (×100). (D) Chimeric gonad during the early stage of sex reversal from ZW females to pseudomale; the green triangle indicates the sperm cell (×100).

Using sex-specific markers (Chen *et al.* 2009), 132 genetic females and 136 males were determined, and, among the genetic males, 66 were determined as pseudomale (Table 3). Normal females (Figure 1A), normal males (Figure 1C), and pseudomales (Figure 1, B and D) in reversal were easily distinguished using tissue sections. Before sex reversal was completed, the ZW fish showed a chimeric gonad; after sex reversal was completed, the gonad developed as that of normal males. Parentage analysis showed that these fish came from 11 families, among which the sires of two families were pseudomales. All the ZW offspring of these two pseudomale families reversed into phenotypic males without any exception.

**Table 3:** The different sex statistics based on parentage analysis

Family	Sire*^a^*	Dam	Female	Pseudomale	Male	Total
1	S1	D10	22	1	22	45
2	S12	D10	3	4	6	13
3	S14	D10	0	10	9	19
4	S16	D15	1	1	4	6
5	S9	D15	0	14	13	27
6	S19	D17	4	3	8	15
7	S38	D3	4	0	6	10
8	S39	D3	2	3	7	12
9	S40	D3	1	3	5	9
10	S2	D7	6	12	16	34
11	S11	D13	23	15	40	78
SUM			66	66	136	268

a Sire S9 and S14 were pseudomales.

### Genome-wide association study

**Figure 2:**
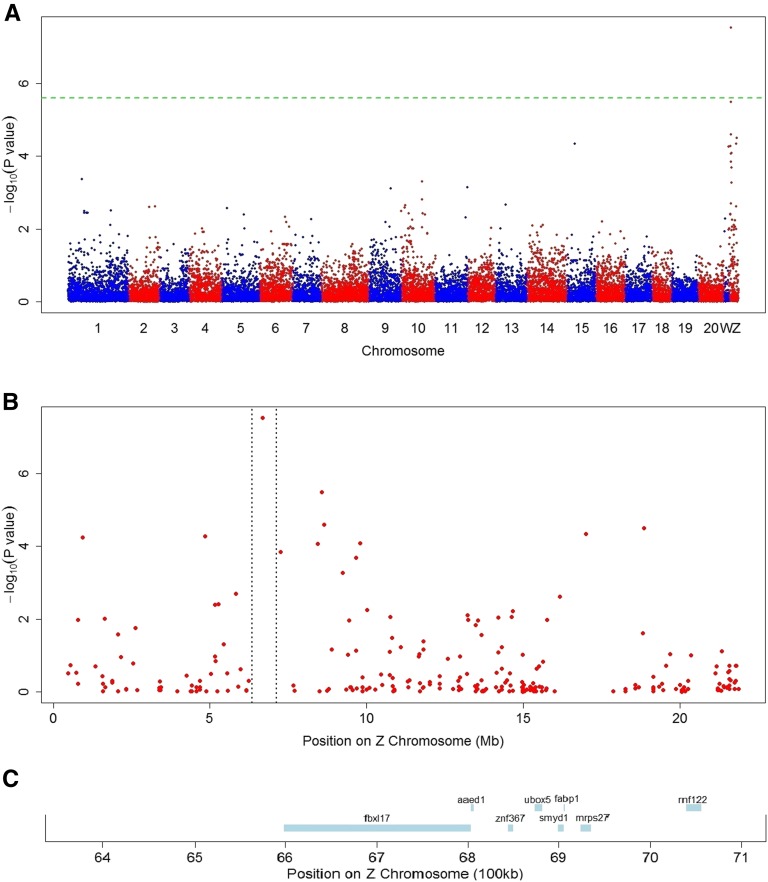
GWAS of sex reversal in the half-smooth tongue sole. (A) Manhattan plot of SNPs associated with sex reversal, *x*-axis presents genomic coordinates along chromosomes 1–20 and sex chromosomes W and Z. The *y*-axis presents a negative logarithm of *P*-values. The horizontal dashed line is the genome-wide threshold, SNPs above this line are significantly associated with sex reversal. (B) Enlarged plot for the Z chromosome, the two vertical dashed lines indicate the region of the 95% confidence interval for the detected locus. (C) The known genes located in the 95% confidence interval.

GWAS was conducted with all the ZW individuals (except for 17 individuals that failed to genotype with 2b-RAD), and the single SNP at 6,676,874 bp on the Z chromosome (named as Cyn_Z_6676874) was found to be strongly associated with sex reversal in half-smooth tongue sole (*P* < 1.0 × 10^−7^; Figure 2A). The SNP Cyn_Z_6676874 was located at the third intron of the F-box and leucine rich repeat protein 17 (*FBXL17*) gene, which is the substrate-recognition component of the SCF (SKP1-CUL1-F-box protein)-type E3 ubiquitin ligase complex, interacting with ubiquitination targets through other protein interaction domains (Jin *et al.* 2004), and the 95% confidence interval was from 6,337,129 to 7,126,693 bp (Figure 2B), where eight known genes were harbored (Figure 2C). Cyn_Z_6676874 explained 82.7% of the genetic variation (the variance components are $\sigma_{g}^{2}=1.36,$ $\sigma_{\text{snp}}^{2}=6.54,$ and $\sigma_{e}^{2}=\left(\pi^{2}/3\right)=3.29,$ respectively), or 58.4% of the phenotypic variation; it had two alleles, A and T, and when the genotype was Z^A^W, none of genetic females reversed, whereas for the Z^T^W genotype, some of the genetic females reversed.

### Confirmation of genetic locus

To confirm SNP Cyn_Z_6676874, we designed primers (Table 4) to amplify the target fragment containing this SNP, and tested it by sequencing in the population sampled in 2014. Among 399 samples, 196 individuals were genetic females, including 95 Z^A^W and 101 Z^T^W genotypes. Tissue sections showed that none of the Z^A^W individuals reversed, whereas for the Z^T^W genotype, 52 individuals reversed into phenotypic males.

**Table 4:** Primers to amplify target fragments

	Sequence (5′–3′)	Length	*T_m_* (°C)	GC (%)
Forward primer	CAGATAGCCAGCACTTAGCCC	21	61	57.14
Reverse primer	CCTGTTGTGAGTGGAGTGTGG	21	61	57.14
Product length	746			

PCR amplification in a volume of 40 μl, containing 20 μl PCR Mix (TaKaRa), 50 ng template, 0.5 μmol/l of each primer. The PCR conditions were: 95° for 3 min, followed by 36 cycles of 94° for 30 sec, 62° for 30 sec, 72° for 1 min, and a final extension step at 72° for 5 min.

## Discussion

Sex reversal has usually been considered an adaptive strategy for animals in evolution (Shapiro 1987; Ghiselin 1974). According Darwin’s theory of evolution, sex reversal must have a genetic basis. The findings in *P. vitticeps* showed that genetic background was related to sex reversal to some extent (Holleley *et al.* 2015), whereas, in the half-smooth tongue sole, it was originally thought that temperature was the critical factor affecting the sex ratio. However, in the present study, the locus Cys_Z_6676874 indicted that the genetic background was the primary basis of sex reversal. This is not in conflict with other reports (S. Chen *et al.* 2014), which showed that higher temperature lead to lower female ratio, because first, our study removed the environmental effects in the experimental design, and it was unnecessary and impossible to repeat earlier experiments; second, we speculated that only Z^T^W individuals were sensitive to temperature, and the female ratio was sensitive to temperature at the population level, especially if the parental genotypes were missing. Our findings suggest that a more detailed experimental design based on the sex reversal locus is important, and necessary, for the further study of temperature-dependent sex determination. Methylation was also thought to cause sex reversal, because the methylation level of pseudomales was higher than that of normal females, and similar to that of normal males (Shao *et al.* 2014). Two pseudomale families also showed possible imprinting occurring for pseudomales, which was inherited by the offspring, because all of them reversed into phenotypic males. Regardless of whether methylation is a cause or a result of sex reversal, the sex reversal locus we detected illuminated the mechanism of sex determination of half-smooth tongue sole.

**Figure 3:**
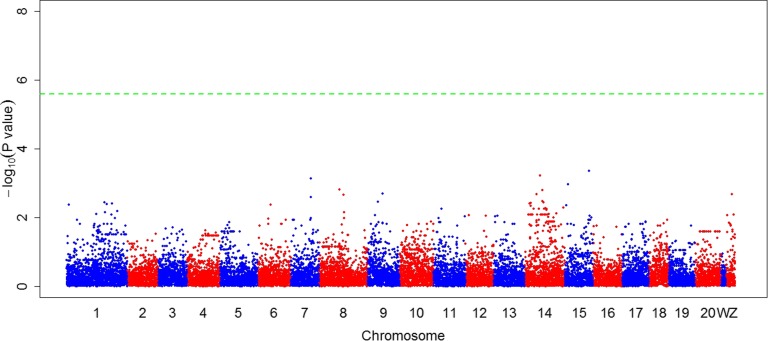
GWAS of sex reversal with the half-smooth tongue sole of the Z^T^W genotype. No locus was detected associated with sex reversal mapping with individuals of the Z^T^W genotype. The green horizontal line is the threshold.

Besides *FBXL17*, there are seven known genes located within the confidence interval of the sex reversal locus: AhpC/TSA Antioxidant Enzyme Domain Containing 1 (*AAED1*), Zinc Finger Protein 367 (*ZNF367*), U-Box Domain Containing 5 (*UBOX5*), SET and MYND Domain Containing 1 (*SMYD1*), Fatty Acid Binding Protein 1 (*FABP1*), Mitochondrial Ribosomal Protein S27 (*MRPS27*), and Ring Finger Protein 122 (*RNF122*). To date, to our knowledge, no reports or obvious evidence have shown that these genes are directly related to various pathways of sex determination. However, they are still worthy of attention because they are located on the sex chromosome, where any variation may have an effect on the genes underlying sex determination. The confirmation of SNP Cyn_Z_6676874 indicated that it was the first barrier regulating sex reversal from genetic females to phenotypic males. We noticed that Z^T^W individuals may not always reverse into phenotypic male; therefore, we performed GWAS again with only individuals of the Z^T^W genotype to determine whether a significant gene interacted with the Cyn_Z_6676874 locus. The results revealed no significant locus (Figure 3), possibly because the sample size was limited, or because minor genes control the remaining 17.3% of the genetic variation of sex reversal, and interact with only the Z^T^W genotype. It is important to increase the sample size of Z^T^W individuals in order to map all loci, and understand the pathway of sex reversal.

**Figure 4:**
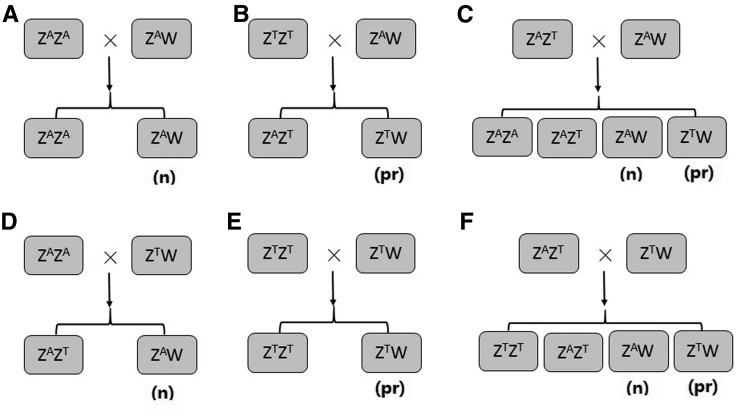
Six possible cross modes of normal males and females. Modes (A) and (B) ensure the genetic female offspring will not reverse to phenotypic males (*n*); in modes (C–F), the genetic female offspring of (Z^T^W) will possibly reverse to phenotypic males (pr). In mode (A), the offspring carries only the A allele, in the next generations, the genetic female will never reverse to the phenotypic male.

Sex reversal has resulted in considerable increased costs in tongue sole aquaculture in recent years in China, and the uncertainty of the female fry ratio has resulted in panic and risk in investment in aquaculture for farmers; therefore, effective management techniques are necessary to control sex reversal and keep the female ratio high. Usually, a specific pseudomale (ZW/male) ratio was considered to improve female percentage in offspring, because pseudomales carry the W chromosome (Piferrer *et al.* 2012). In our study, we did not find the Z^A^W/female×Z^T^W/male cross, but the offspring from two Z^T^W/female×Z^T^W/male families precluded this hypothesis, and verified its impossibility in aquaculture, similarly to the conclusion of other studies (L. Chen *et al.* 2014). The sex reversal locus reported here could be used directly in breeding to produce nonreversed half-smooth tongue soles, keeping the phenotypic female ratio of offspring at around one-half. Using Z^A^Z^A^ males as sires, the genetic female offspring are Z^A^W, and none of them will reverse into phenotypic males (Figure 4, A and D). If normal female Z^A^W are used, then the unfavorable allele T will be removed from the population, and the genetic female offspring of the next generations will never reverse into phenotypic males (Figure 4A). These two mating techniques are beneficial for the effective management of the aquaculture of the half-smooth tongue sole.

## Supplementary Material

Supplemental material is available online at www.g3journal.org/lookup/suppl/doi:10.1534/g3.116.036822/-/DC1.
