# Supplementary material for: Single Locus Maintains Large Variation of Sex Reversal in Half-Smooth Tongue Sole (Cynoglossus semilaevis)
Source: G3 (Bethesda). 2016 Dec 21;7(2):583–9. doi: 10.1534/g3.116.036822 (PMC5295603; doi:10.1534/g3.116.036822)
Supplement: Supplementary file 7 [file 583FileS4.docx]

File S4. SNP information. (.txt, 323 KB)

<http://www.g3journal.org/lookup/suppl/doi:10.1534/g3.116.036822/-/DC1/FileS4.txt>
